# Role of Adiponectin in prostate cancer

**DOI:** 10.1590/S1677-5538.IBJU.2018.0261

**Published:** 2019-04-01

**Authors:** Xiaobo Hu, Cong Hu, Caiping Zhang, Min Zhang, Shiyin Long, Zhaohui Cao

**Affiliations:** 1 Hunan Province Cooperative Innovation Center for Molecular Target New Drug Study, University of South China, Hengyang, China; 2 Department of Biotechnology, School of Pharmacy and Biosciences, University of South China, Hengyang, China

**Keywords:** Prostatic Neoplasms, Obesity, Stress, Physiological

## Abstract

Obesity is defined as a chronic and excessive growth of adipose tissue. It has been associated with a high risk for development and progression of obesity-associated malignancies, while adipokines may mediate this association. Adiponectin is an adipose tissue-derived adipokines, with significant anti-diabetic, anti-inflammatory, anti-atherosclerotic and anti-proliferative properties. Plasma adiponectin levels are decreased in obese individuals, and this feature is closely correlated with development of several metabolic, immunological and neoplastic diseases. Recent studies have shown that prostate cancer patients have lower serum adiponectin levels and decreased expression of adiponectin receptors in tumor tissues, which suggests plasma adiponectin level is a risk factor for prostate cancer. Furthermore, exogenous adiponectin has exhibited therapeutic potential in animal models. In this review, we focus on the potential role of adiponectin and the underlying mechanism of adiponectin in the development and progression of prostate cancer. Exploring the signaling pathways linking adiponectin with tumorigenesis might provide a potential target for therapy.

## INTRODUCTION

Prostate cancer (PC) recently became the second most prevalent cancer afflicting men, and the fifth leading cause of cancer related death throughout the World (1, 2). Age, familial history, smoking, sedentary lifestyle and overweight are all factors in the pathogenesis of PC. Of note, obesity is well known as an increased risk for several cancers (including colon, ovary, breast, esophagus and pancreatic), also for PC (3, 4). The links between obesity and PC are complicated. Three possible mechanisms are proposed to help explain the association between obesity and the increased risk of PC: the insulin / insulin-like growth factor-1 (IGF-1) axis, sex hormones and adipokines signaling (5, 6). Adiponectin (APN), an adipocyte-secreted adipokine, operates in the maintenance of many physiological functions, having potential benefits in the prevention of certain diseases. It mainly regulates inflammation and influences glucose and lipid metabolism through its insulin sensitizing effects (7, 8). Recently, APN was proved to be one of the mediators in the development and progression of several types of obesity-associated cancers (9). In this report, we summarized recent findings on the potential role of APN and the underlying mechanism of APN in PC. In addition, the clinical values of APN for PC patients will also be highlighted.

### Adiponectin and its receptors

Adiponectin, also called Acrp30, is a 28-30-kDa adipokine produced mainly by adipose tissue. Full-length APN (fAPN) consists of an N-terminal signal sequence, a short hypervariable region, a collagen-like domain and a C-terminal globular domain (10, 11). Pre-secretion, posttranslational processing generates trimers, hexamers, and high molecular weight (HMW)-APN. HMW-APN appears to be the physiologically most relevant and dominant form in plasma (10). Fruebis et al. (11) identified the fourth fraction of APN in the plasma, the globular domain of APN (gAPN), which is generated by the proteolytic cleavage of fAPN.

Normal plasma APN levels range from 5 to 30 µg / mL, accounting for up to 0.05% of total plasma proteins in humans (11). Despite the fact that APN is produced mainly by adipose tissue, its serum concentration is reversely correlated with the body mass index (BMI) (12). One possible explanation of the reduced APN levels in obesity may be caused by the enhanced production of proinflammatory cytokines, in particular, by TNF-α, IL-6. Another explanation that serves to downregulate APN expression is endoplasmic reticulum (ER) stress resulting from obesity. In addition, it is demonstrated that there is a negative feedback of APN on its own production during the development of obesity (12). APN plasma concentration was found to be sex dependent, with there being a higher serum concentration in women than in men (12).

Although produced by adipose tissue, APN functions via the specific receptors, AdipoR1 and AdipoR2. Both these receptors contain seven transmembrane domains with the C-terminus inside the cells and the N-terminus outside (13). Specifically, AdipoR1, ubiquitously expressed and highly expressed in skeletal muscle, possess a high-affinity for gAPN and a low affinity for fAPN. In contrast, AdipoR2, predominantly expressed in the liver, binds both gAPN and fAPN with an intermediate affinity (13). T-cadherin has been successively discovered as a third receptor for APN, with a high affinity to HMW-APN (14). It is an atypical glycosyl phosphatidylinositol-anchored cadherin, located on the cellular surfaces of endothelial, epithelial, and smooth muscle cells (14). Because T-cadherin lacks both transmembrane and cytoplasmic domains, it is considered to have no effect on APN cellular signaling or function. Its main role is thought to act as an APN-binding protein, rather than a receptor.

### Adiponectin and prostate cancer

Adiposity has been consistently associated with an increased risk of progression of PC, but APN is inversely related to the degree of adiposity. It seems that plasma APN should be reduced in PC patients. Goktas et al. (15) was the first to report that serum APN levels were significantly lower in patients with PC than in the BPH group or in healthy controls. In addition, APN levels were negatively associated with histological grade and disease stage. Next, a study of 300 Greek men by Michalakis et al. (16) revealed a significantly reduced risk of PC with higher plasma APN concentrations. In line with this, APN receptor levels are likewise lower in resected PC tissues. Several studies have supported the inverse association between APN and risk of PC or high-grade PC (17-21). Therefore, these findings indicated the potential role of APN in the suppression of carcinogenesis. However, as HMW-APN is the most active form of APN, Medina EA et al. (22) evaluated the relation between HMW-APN and PC, and found that only HMW-APN decreased the risk of PC in obese man. In 2006, Baillargeon et al. (23) found no correlation between APN levels and the development of PC. Furthermore, they found no association of PC with BMI, leptin and IL-6. In 2014, a study by Stevens et al. (24) also revealed APN is not associated with risk of aggressive PC. Thus, the precise role of APN in the development of PC remains elusive. In 2015, using a cross-sectional study, Ikeda A et al. (25) detected PC by prostate-specific antigen (PSA)-based screening and firstly demonstrated a significant and positive correlation between APN levels and PSA levels. One explanation was the proportion of obese men in the study was extremely low, another one was maybe the increased APN being a protective response against tumor progression, and the third one was they did not evaluate HMW-APN in this study. Finally, Liao Q (26) performed a meta-analysis of numerous studies and concluded that patients with PC markedly had lower APN levels than controls, they also found that decreased concentration of APN was associated with a significantly greater risk of PC.

Given that APN encoding gene (ADIPOQ) and its receptor (ADIPOR) are highly polymorphic and carry several single nucleotide polymorphisms (SNPs), the genetic variants of ADIPOQ and ADIPOR might affect PC risk. In a study with 1,286 cases and 1,267 controls, Dhillon P et al. (27) found four SNPs of ADIPOQ were significantly associated with PC risk, two of which were also associated with plasma APN concentration. Furthermore, Kaklamain et al. (28) conducted a study that also noted the effects of genetic variations in ADIPOQ and ADIPOR1 gene on PC. Conversely, two other studies demonstrated no associations in candidate SNPs and PC risk (29, 30). Since these results were inconsistent and insufficient, an updated meta-analysis performed by Hu et al. revealed that ADIPOQ rs 2241766 and ADIPOR1 rs 10920531 variants were identified to be correlated with increased risk of PC. On the contrary, ADIPOR1 rs 2232853 variants were associated with decreased risk of PC (31). The identified polymorphisms might help prediction of prevalence and prognosis of PC, as well as generation of novel targeted therapies.

The relationship between APN receptors and PC has also been examined by many investigators. Mistry T et al. (32) firstly showed AdipoR1 and AdipoR2 expressed both in benign and adenocarcinomatous prostate tissue by immunohistochemistry analysis. The presence of the receptors expression suggested that APN binding to its receptors may mediate prostate physiology and pathologic changes. In 2007, Michalakis K et al. (16) showed that malignant prostate tissue samples reduced expression of APN receptors compared with benign prostate tissue. In line with APN receptors, the serum APN concentrations in patients with PC were lower than those in controls, which led to the hypothesis that receptor downregulation in PC would promote cancer progression. However, another study conducted by Rider JR et al. (33) revealed that there was a positive relationship between AdipoR2 and PC development. The relationship between APN receptors expression and carcinogenesis is somewhat controversial, therefore more studies are needed to elucidate the link between APN receptors and PC. The results of epidemiological studies of the association between APN levels and PC are summarized in Table-1.

**Table 1:** Recent Studies showing the association between APN concentrations and risk of PC.

Reference	Sample numbers	APN levels / OR	Comments / Conclusion	Other findings	TS
Goktas S (15)	30 PC	5.3 ± 1.6 µg / mL	APN concentrations are lower in PC than BPH or in control subjects	APN are negatively associated with the histologic grade and disease stage of PC	CC
	41 BPH	14.5 ± 4.4 µg / mL			
	36 Con	16.2 ± 4.1 µg / mL			
Michalakis K (16)	75 PC	7.4 ± 5.0 µg / mL	Higher plasma APN concentrations are associated with a reduced risk of PC	AdipoR1 and AdipoR2 in cancerous were weaker expressed compared with healthy prostate tissue	CC
	75 BPH	11.5 ± 6.4 µg / mL			
	150 Con	12.8 ± 8.0 µg / mL			
Schenk JM. (17)	698 BPH	OR = 0.43	High APN concentrations were associated with reduced risk of BPH	Neither C-peptide nor leptin was associated with BPH risk	NCC
	709 Con				
Li H (18)	654 PC	Q1: 2.7 µg / mL	Higher APN concentrations have a lower risk for developing high-grade or metastatic cancer	Leptin was unrelated to PC risk or mortality	NCC
	644 Con	Q3: 6.3 µg / mL			
		Q5: 13.3 µg / mL			
Tan W (19)	96 PC	Low level of APN	APN was significantly decreased in PC compared with that of BPH tissues	APN may function as a tumor suppressor through inhibiting EMT of PC cells.	CC
	15 BPH	BPH: 1of 15 (6.7%) GS <7: 6of 27 (22%)			
		GS = 7: 18of 26 (69%)			
			Decreased APN level was significantly associated with high GS		
		GS > 7: 32of43 (74%)			
Medina EA (22)	228 PC	OR = 0.62	Only HMW APN decreased the risk of PC in obese man	HMW increased the risk of PC in normal and overweight men	NCC
	239 Con				
Baillargeon J (23)	125 PC	17.9 ± 0.6 µg / mL	APN was not significant associated with PC risk	BMI was not associated with incident PC	NCC
	125 Con	19.9 ± 13.2 µg / mL			
Stevens VL. (24)	272 PC	OR = 1.11	APN was not associated with risk of aggressive PC	C-peptide was not associated with risk of aggressive prostate cancer	NCC
	272 Con				
Ikeda A. (25)	24 PC	9.96 µg / mL	APN was significantly and positively associated with PSA levels	High APN increased the Incidence of low-or mediate-risk PC in obese man	CS
	2817 Con	7.64 µg / mL			

**APN** = adiponectin; **PC =** prostate cancer; **BPH =** benign prostatic hyperplasia; Con = Control; **TS =** type of study; **CC =** case - control; **NCC =** nested-case-control; **CS =** cross-sectional; OR = odds ratio; Q5 = Highest quintile; **Q3 =** intermediate Quintile; **Q1 =** Lowest Quintile; **GS =** Gleason score; **PSA =** prostate-specific antigen.

### Potential mechanisms of adiponectin in prostate cancer

Recent advances suggested that APN plays a role in carcinogenesis through numerous mechanisms including inhibiting proliferation and inducing apoptosis (19, 20, 34-42). Recent studies have shown that activation of the adenosine monophosphate-activated protein kinase (AMPK) is a key part of the signaling cascade downstream of APN receptor (20, 34-36). The proteins downstream of AMPK included tuberous sclerosis protein 2 (TSC2), the mammalian homologue of the target of rapamycin (mTOR), vascular endothelial growth factor A (VEGF-A) and fatty acid synthase (FAS), all of which are involved in the regulation of cell proliferation. In PC-3 cells, activation of AMPK by APN is associated with reduction in mTOR activation, which reduces protein translation and inhibits cell growth (34). In this study, when siRNA reduced AMPK level, APN-induced growth is significantly inhibited.

Another study of modification of APN levels in PC-3 cells supported that APN activates AMPK / TSC2 to inhibit mTOR-mediated VEGF-A activation and to inhibit cancer neovascularization (20, 35). In addition, AMPK prevents fatty acid synthesis by downregulation FAS, then inhibited cell growth and induced apoptosis in LNCaP cancer cells (36).

Signal transducers and activator of transcription 3 (STAT3) appears to be a key regulator for cell proliferation and apoptosis. Both fAPN and gAPN can stimulate JNK activation, then drastically suppress STAT3 activation in DU145 cells, suggesting that JNK and STAT3 may constitute a universal signaling pathway to mediate APN's pathophysiological effects on PC (37).

APN also has anti-proliferation effects on many cell lines including PC3, DU145 and LNCaP PC cells (38, 39). APN induced cell cycle arrest of prostatic epithelial and stromal cell lines through the downregulation of cyclinD1 and PCNA, which attenuated the growth factor-mediated proliferation. Additionally, APN induced apoptosis by increasing caspase 3, Bax expression and downregulation of Bcl2 (40).

Furthermore, APN significantly inhibits cell proliferation induced by leptin. Different leptin / adiponectin ratios resulted in varying inhibitory effects on PC cells, indicating the balance between APN and leptin might effectively modulate PC cell growth. APN can attenuate the adverse effects of leptin and inhibit LNCaP and PC3 proliferation via modulation of p53 and bcl-2 expression (41), hence the balance of leptin and APN may be important in driving obesity-related PC progression.

**Figure-1:**
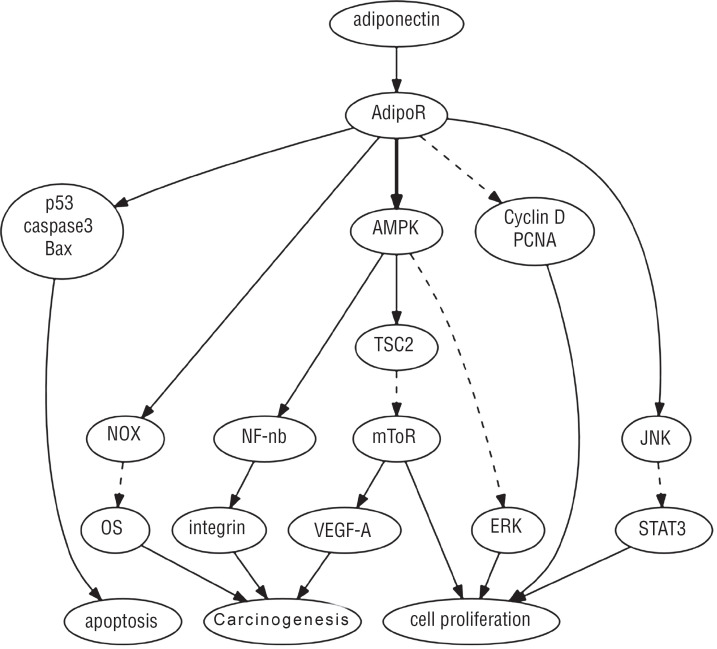
Signaling pathways of adiponectin in prostate cancer cells. **JNK =** c-Jun N-terminal kinase; **STAT3 =** signal transducer and activator of transcription; **AMPK =** AMP-activated protein kinase; **TSC2 =** tuberous sclerosis complex 2; **mTOR =** mammalian target of rapamycin; **NF-KB =** nuclear factor-KB; **NOX =** NADPH oxidase; **OS =** oxidative stress; ↓ indicates stimulation; 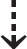 indicates inhibition.

Oxidative stress (OS) is a key event in the initiation, development and progression of PC. APN increased cellular anti-oxidative defense mechanisms and inhibited OS via increasing NADPH oxidase NOX2 and NOX4 expression in human 22Rv1 and DU-145 PC cell lines (42). Despite an increasing accumulation of experimental data, the mechanisms underlying the anti-proliferative and tumor-suppressing effects of APN are still not fully understood, more studies are needed. The role of APN in PC is summarized in Figure-1.

### Clinical values of Adiponectin for prostate cancer patients

Based on the signaling pathway conducted by APN and its receptors, APN might represent a promising therapeutic target. Currently, the administration of APN or direct antagonist has not been reported in the literature for the treatment of human cancers. A strategy for the future treatment of PC patients with hypoadiponectinemia may include the upregulation of APN levels, APN receptors, or the development of APN receptor agonists.

Tangible benefits may come from the anti-diabetic drug Metformin. Metformin partially mimicked APN action and activate AMPK signaling in PC-3 cells, with reduction of mTOR activity thus inhibition of cell growth, suggesting that Metformin might have particular value in attenuating the adverse effects of hypoadiponectinemia on PC (34). Metformin therapy has been correlated with reduced risk of prostate cancer in Caucasian / white men with diabetes (43) and with a survival beneﬁt after diagnosis (44). These results underscore the importance for further studies to evaluate metformin in PC. Plasma APN levels can also be upregulated by thiazolidinediones (TZDs), such as pioglitazone, rosiglitazone, a class of PPAR-γ agonists and medicine used in the treatment of type II diabetes (T2D). Clinically, Metformin and thiazolidinedione therapy improved survival of diabetics prostate cancer patients (45).

Down-regulation of APN in PC tissues and LNCaP cells owing to highly methylation in its promoter, and 5-AZA restored its expression in vitro. Thus, methylation of APN promoter may be a key factor for evaluation of PC, and 5-AZA may be a promising stimulator of APN (19). Zorn CS et al. (46) reported that 5-AZA improves survival in the transgenic adenocarcinoma of the mouse prostate (TRAMP) model.

Previous research suggested that diet pattern as well as physical activity might increase expression of APN and delay disease progression in PC patients (47, 48). Hence, moderate physical exercise, reduction of body fat, associated with restriction of calories in diet are recommended for obesity-related prostate cancer prevention.

Another potential therapeutic molecule is the APN receptor agonist. Agonists have been developed and tested to treat multiple diseases related to hypoadiponectinemia, diabetes and other malignancies (49, 50). ADP355, a first-in-class APN receptor agonist, restricted proliferation in several APN receptor-positive cancer cell lines, and suppressed the growth of established tumors by 31% in vivo (49). AdipoRon, with similar effects to APN, increased apoptosis while inhibiting pancreatic cell proliferation and colony formation. In vivo, treatment of mice with AdipoRon inhibits orthotopic pancreatic tumor growth (50). APN receptor agonists may represent novel therapeutic strategies for PC in future.

## CONCLUSIONS

Numerous studies supporting the notion that APN acts as a protective and safe factor to prevent progression of PC, but few studies may indicate otherwise. We summarized the mechanisms underlying the anti-proliferative and tumor-suppressing effects of APN specifically in PC without reiterating other types of cancers. The signaling pathways linking APN with tumorigenesis involve several key molecules, including AdipoRs, AMPK, JNK, NOX, NF-κB, and so on, thus providing potential drug targets for the future. Based on the beneficial effects induced by APN, future efforts can focus on increasing APN and its receptors levels in response to PC. Since APN exerts pleiotropic effects on different tissues and exists as a high serum concentration protein, the main role of APN should be regulation of metabolism, not necessarily to act as an anti-cancer hormone. We must consider many other risk factors for PC with a lower level of APN, which could help develop better approaches for the treatment and prevention of all men with PC. Moreover, further research is warranted to better understand the pathophysiological role of APN in obesity and obesity-related PC, and elucidate the potential clinical application in humans.
